# Association between matrix metalloproteinase-9 gene polymorphism and breast cancer in Brazilian women

**DOI:** 10.6061/clinics/2020/e1762

**Published:** 2020-10-21

**Authors:** Victor Alves de Oliveira, Diego Cipriano Chagas, Jefferson Rodrigues Amorim, Renato de Oliveira Pereira, Thais Alves Nogueira, Victória Maria Luz Borges, Larysse Maira Campos-Verde, Luana Mota Martins, Gilmara Peres Rodrigues, Elmo de Jesus Nery, Fabiane Araújo Sampaio, Pedro Vitor Lopes-Costa, João Marcelo de Castro e Sousa, Vladmir Costa Silva, Felipe Cavalcanti Carneiro da Silva, Benedito Borges da Silva

**Affiliations:** I Programa de Pos-Graduacao em Ciencias e Saude, Universidade Federal do Piaui, PI, BR; II Programa de Doutorado em Biotecnologia Rede Nordeste de Biotecnologia (RENORBIO), Universidade Federal do Piaui, PI, BR

**Keywords:** MMP9, Breast Cancer, Risk, rs17576, SNP Variants

## Abstract

**OBJECTIVE:**

This study aimed to determine the relationship between rs17576 (MMP-9) polymorphism and increased cancer risk in a Brazilian breast cancer cohort.

**METHODS:**

This study included 141 women (71 breast cancer patients and 70 controls without breast cancer) who donated 3 mL of their peripheral blood for genomic DNA extraction. This DNA was then genotyped using a real-time polymerase chain reaction.

**RESULTS:**

The AG (rs17576) genotype was identified in 26 (18.43%) participants in the case group and in 22 (15.60%) participants in the control group (*p*=0.274), while the GG genotype was identified in ten (7.09%) participants in the case group and in one (0.70%) participant in the control group (*p*<0.003 - OR (95% CI) 13.13 (1.73, 593.08). No significant difference in the incidence rates was observed for AG or GG rs17576 genotypes in premenopausal women, *p*=0.813 and *p*=0.556, respectively. However, in postmenopausal women, the AG genotype was shown to occur in 14 (22.5%) participants in the case group and in 4 (6.45%) participants in the control (*p*<0.043), while GG genotype occurred in eight (12.90%) of the individuals in the case group and in none of the individuals in the control group (*p*<0.006).

**CONCLUSION:**

In this study, the MMP-9 rs17576 GG polymorphic variant was shown to be significantly associated with breast cancer risk in premenopausal women, while the AG and GG genotypes were associated with increased cancer risk in postmenopausal women.

## INTRODUCTION

Breast cancer is the second most common malignant neoplasm in the female population of Brazil after non-melanoma skin cancer, with an estimated 59,700 new cases and 14,206 deaths in 2018 (1). Breast neoplasm is a multifactorial disease of unknown etiology, in which diverse factors can drive increased cancer risk. Genetic factors are responsible for a subset of hereditary cases and for the development and progression of this disease (2).

Variations in the expression of matrix metalloproteinases (MMPs) have been linked to increased risk of developing breast cancer. MMPs are a family of zinc-dependent proteinases that are all structurally and physiologically related based on their ability to degrade the extracellular matrix (ECM) (3). *In vivo*, MMP activity in the extracellular environment has been linked to the progression of various malignancies, including breast cancer. MMPs may exert both direct and indirect effects on tumor progression. One of the examples of their direct effects is the promotion of tumor cell proliferation and metastatic dissemination, while the promotion of angiogenesis—a phenomenon responsible for providing nutrition to tumors, thereby contributing to their dissemination—is an example of their indirect effect (4,5).

The matrix metalloproteinase-9 (*MMP-9*) gene encodes an enzyme that hydrolyzes ECM components and assumes a primordial function during tissue remodeling, resulting in angiogenesis and metastases (6). Some phenomenon, including single nucleotide polymorphisms (SNPs), the most prevalent form of genetic variation in the human genome, may alter the expression and activity of MMP-9 (7). The polymorphic MMP-9 SNP rs17576 is a missense mutation that results in the substitution of glutamine with Arginine, resulting in alteration of the three dimensional conformation and activity of MMP-9; the mutant enzyme can promote basement membrane degradation, neovascularization, and tumor metastasis (8-10). However, several other studies have linked this variant as protective in cancer, making the role of this variant somewhat controversial (6). Despite this there is a paucity of studies on this SNP in the literature, therefore, we investigated this SNP in this study.

The link between rs17576 and breast cancer susceptibility remains unclear, therefore this study was designed to evaluate the relationship between this polymorphism and increased cancer risk in a Brazilian breast cancer cohort. 

## MATERIAL AND METHODS

### Study Population

This controlled cross-sectional study evaluated the genotype of 141 women, recruited from the Outpatient Clinic at the Getúlio Vargas Hospital, Federal University of Piauí, Teresina, Piauí, Brazil. Patients in the case group were recruited throughout 2014 and the participants in the control group were recruited from July 2015 to June 2016. The study design was based on a minimum participant number of 66 per group to enable the evaluation of valid polymorphisms. Among the patients, 71 had histologically confirmed breast cancer and were randomly selected for the case group; 70 healthy women—confirmed by physical examination and mammography as being negative for breast malignancy—were recruited as age-matched controls (±3 years). Patients were excluded from the study if they were affected by any other type of cancer or if they were over 80 years old. All study participants signed an informed consent prior to the initiation of our investigations. Our research protocol complied with the Resolution 466/2012 proposed by the Brazilian National Health Council and was approved by the Research Ethics Committee at the Federal University of Piauí, Teresina, Brazil (CAAE 43447015.8.0000.5214). This study was compliant with the terms established in the Declaration of Helsinki (1964) and its later amendments or other comparable ethical standards.

### Blood Collection and DNA Extraction

A 3 mL aliquot of whole peripheral blood was collected and stored in appropriate bags containing (EDTA) anticoagulant solution at -20°C. Genomic DNA was extracted from 200 µL of whole blood, using the DNeasy^®^ Blood&Tissue Kit (QIAGEN^®^), as per the manufacturer’s instructions. Qualitative and quantitative estimation of the DNA in each sample was performed using a Nanodrop 2000 spectrophotometer (Thermo Fisher Scientific, Waltham, MA, USA). The concentration of all DNA samples was adjusted to 20 ng/µL before genotyping and the DNA samples were stored at -20°C for long-term storage.

### Genotyping

Genotyping was performed in the Molecular Biology laboratory at the Institute of Tropical Diseases under the guidance of Dr. Natan Portela, Teresina, Piauí, Brazil, using real-time polymerase chain reaction (RT-PCR). Amplification was performed on the Fast RT-PCR System 7500 with SDS 2.2 software for SNP genotyping (Thermo Fisher Scientific, USA).

Reactions were standardized in a final volume of 20 μL and PCR was performed in triplicate. Each reaction contained 10 µL of the TaqMan^®^ Genotyping Master Mix; 0.5 µL of the TaqMan^®^ custom probe for SNP genotyping of human *MMP*-9 (SNP ID rs17576. Cod. C__11655953_10) (Table 1); 5.5 µL of DNA/RNA-free water; and 4 µL of DNA per patient. Fluorescence data were captured over a 45 cycle program and the quality of the RT-PCR was evaluated by random sampling which was subject to re-genotyping.

### Statistical Analysis

*t*-test was used to compare the age of the groups and subgroups. Chi-square test was used to determine whether genotype distribution was in agreement with the Hard-Weinberg equilibrium and the genotype frequency was compared between breast cancer patients and the control group (healthy women), using a Fisher’s exact test as described by Agresti & Kateri (11). The odds ratio (OR) and 95% confidence interval (CI) were calculated using a Fisher’s exact test because of the low frequency of several of the variations. A Pearson's test was used to assess the correlation between various clinical features and the *MMP-9* rs17576 genotype. Significance was set at *p*<0.05.

## RESULTS

This study included 141 women (71 cases and 70 controls) and the mean patient age in the cancer group was 49.9±12.03 years (27/76), while in the control group the mean age was 45.3±12.77 years (18/78). No significant difference was observed between the mean ages of any of the groups or subgroups evaluated in this study (Table 2). The genotype frequency for MMP-9 rs17576 was in agreement with the Hardy-Weinberg equilibrium, with the AG genotype being identified in 26 patients (18.43%) in the case group and in 22 patients (15.60%) in the control group (*p*=0.2746), while the GG genotype (rs17576) was identified in ten (7.09%) women in the case group and in one (0.70%) woman in the control group (*p*<0.0030 - OR (95% CI) 13.13 (1.73, 593.08)) (Table 2).

After stratification based on menopausal status, the AG (OR=1.14; 95%CI: 0.40-3.23; *p*=0.8132) and GG genotypes (OR=3.15; 95%CI: 0.15-196.26; *p*=0.5561) were not found to be significantly associated with breast cancer risk in premenopausal women when compared to wildtype (AA). Conversely, there was a significant difference in AG and GG genotype frequencies in postmenopausal women with and without breast cancer, *p*<0.043 and *p*<0.0065, respectively (Table 3).

There were no significant associations between the clinical features of breast cancer (tumor staging; presence of metastasis; compromised lymph nodes; lymphatic invasion; blood invasion; estrogen receptor; progesterone receptor; and recurrence) and the genotypes identified in these groups (*p*>0.05) (Appendix - Supplementary Table 1).

## DISCUSSION

Currently, 36 SNPs and 30 clinically important variants have been described for *MMP-9* (12). Liu et al. (13) reported that the rs17576 variant is a possible prognostic marker for overall survival in patients with locoregionally advanced nasopharyngeal carcinoma treated with chemoradiotherapy. Although the results have varied, a few studies have reported an association between this variant and increased breast cancer risk (14-17).

In this study, we evaluated the association between *MMP-9* rs17576 polymorphisms and breast cancer risk in a Brazilian cohort. Our results showed a significant difference in the incidence of the GG genotype in breast cancer patients and identified an increased risk of breast cancer in postmenopausal women harboring either the AG or GG genotype at this locus.

In a study on 245 Chinese women with invasive breast cancer, Fu et al. (14) showed no correlation between *MMP-9* rs17576 variant and survival free from locoregional disease and distant metastasis. Similarly, in a meta-analysis review, including ten articles evaluating *MMP-9* polymorphic variants, Zhang et al. (17) found that the *MMP-9* rs17576 polymorphism had been evaluated in only three studies and had demonstrated no association between this SNP locus and changes in breast cancer risk.

Conversely, Chahil, et al. (16) studied the association between SNPs and breast cancer risk and found a significant correlation between breast cancer risk and three SNPs, including the *MMP-9* rs17576 GG variant, which we investigated in this study. Resler et al. (15) conducted a case-control study and investigated 233 SNPs from 31 genes in 845 invasive breast cancer cases and 807 controls. This study revealed a significant association between rs17576 polymorphic variants and increased breast cancer risk.

Conflicting results for this gene polymorphism have been described for other tumors, including prostate and urinary tract cancers. Some findings revealed that rs17576 acts as a protective polymorphism while other studies demonstrated its association with increased cancer risk, but many of these studies are controversial and inconsistent because of the small number of studies available (18,19). Kin et al. (20) evaluated 326 patients with chronic gastritis and 153 patients with gastric cancer, 59 patients with lymph node metastasis, and found no association between the presence of rs17576 polymorphisms and the development of gastric cancer or lymph node metastasis.

However, the association between MMP-9 SNPs and gastric cancer has been established in the Chinese population, especially rs17576 and rs2250889 SNPs are significantly associated with lymph node metastases, especially in diffuse type gastric cancer. This study used 174 patients, 74 cases stratified into lymph node metastasis (n=45) and no lymph node metastasis (n=29) (21). Okada et al. (10), studied 4434 individuals from the Japanese population and demonstrated that the rs17576 (MMP-9) polymorphism may be a functional mutation associated with a multiple family history of gastric cancer.

It is noteworthy that there is evidence indicating that MMP-9 (rs17576) genotypes and haplotypes affect MMP-9 expression in obese children and in patients of African descent, suggesting that they may exert different effects, resulting in different and varied pathologies (7,22). Moreover, Štrbac et al. suggest that serum MMP9 levels and polymorphisms should be studied as possible diagnostic and prognostic biomarkers for malignant mesothelioma (23).

Despite the controversial data in the literature regarding the association between rs17576 and breast cancer and other types of cancer, this study sought to evaluate the role of this SNP in the Brazilian population. This study is limited by the small number of studies and patients analyzed, which makes it more difficult to better characterize the involvement of the rs17576 locus in cancer risk, especially in breast cancer patients.

## CONCLUSION

In summary, the results of this study show a significant association between MMP-9 rs17576 (GG) genotype and breast cancer risk. In addition, AG and GG genotypes of MMP-9 rs17576 are both associated with increased cancer risk in postmenopausal women. There is a need to validate these findings in a larger cohort, especially in a country known for its genetic diversity.

## AUTHOR CONTRIBUTIONS

V.A. Oliveira, J. R. Amorim, D.C. Chagas, R.O. Pereira, L.M. Campos-Verde, T.A. Nogueira, V.M.L Borges, L.M. Martins, G.P. Rodrigues, E.J.N. Júnior, F.A. Sampaio, P.V. Lopes-Costa, J.M.C. Sousa, V.C. Silva, F.C.C. Silva, and B.B. da-Silva contributed to the conceptualization, data curation, formal analysis, investigation, methodology, project administration, resources, supervision, validation, visualization, writing-original draft, and writing/reviewing and editing.

## Figures and Tables

**Table 1:** Description of the SNP TaqMan^®^ assays used in this study.

Gene Code	SNP Location	Context sequence VIC/FAM	SNP Type	Change in Codon
MMP 9	885	CTCCTCGCCCCAGGACTCTACACCC**[A/G]**GGACGG	Missense	CAG/CGG
rs17576 A>G		CAATGCTGATGGGAAACCC		

Source: Thermo Fisher Scientific.

**Table 2:** Patient characteristics and rs17576 genotype for both case and control patients.

Characteristics		Case	Control	*p*
N		71	70	
Mean age (SD)		49.99 (±12.03)	45.38 (±12.77)	0.071
Premenopausal		40	46	
Mean age (SD)		49.46 (±10.42)	47.13 (±13.07)	0.372
Postmenopausal		31	24	
Mean age (SD)		48.34 (±11.99)	42.04 (±11.71)	0.055

	Case (%)	Control (%)	*p*	OR (95% CI)
rs17576				
AA	35 (24.82)	47 (33.33)		
A/G	26 (18.43)	22 (15.60)	0.2746	1.58 (0.73, 3.46)
GG	10 (7.09)	1 (0.70)	0.0030	13.13 (1.73, 593.08)

A significant difference was observed in the incidence of the rs17576 GG genotype between the case and control groups (*p*<0.003). The odds ratio (OR) was significantly higher in the case group.

**Table 3:** Genotyping of rs17576 in premenopausal and postmenopausal patients.

	Premenopausal				Postmenopausal			
Genotype	Case	Control	*p*	OR (95% CI)	Case	Control	*p*	OR (95% CI)
A/A	18	29	-	-	17	19	-	-
A/G	12	17	0.8132	1.14 (0.40, 3.23)	14	4	0.0431	3.81 (0.95, 19.07)
G/G	2	1	0.5561	3.15 (0.15, 196.26)	8	0	0.0065	-

A significant difference was observed in the incidence rates of rs17576 AG and GG genotypes between case and control groups of postmenopausal women (*p*<0.05).

## APPENDIX

**Supplementary Table 1:** Correlation between rs17576 genotype (MMP-9) and clinical features.

Features		Alleles			*p*
		AA	AG	GG	
Stage	1	5	3	4	0.966
	2	19	15	1	
	3	10	7	5	
Metastasis	Yes	10	10	2	0.928
	No	25	16	8	
Lymph node metastasis	Yes	14	11	5	0.605
	No	21	15	5	
Lymphatic invasion	Yes	15	10	2	0.237
	No	20	16	8	
Blood vessel invasion	Yes	4	2	0	0.265
	No	31	24	10	
Estrogen receptor	Negative	6	9	2	0.446
	Positive	28	18	8	
Progesterone receptor	Negative	9	9	2	0.987
	Positive	26	17	8	
Recurrence	Yes	18	12	2	0.117
	No	17	14	8	
